# Pharyngeal electrical stimulation for neurogenic dysphagia of different aetiologies

**DOI:** 10.1016/j.eclinm.2020.100626

**Published:** 2020-11-19

**Authors:** Domenico A. Restivo

**Affiliations:** a Department of Medicine, Neurological Unit, “Garibaldi” Hospital, Catania, Italy; b School of Specialization in Rehabiliation Medicine, University of Padova, Italy

Neurogenic dysphagia can occur in patients with neurological disorders of different etiologies, and is associated with poor long-term outcome, high mortality, morbidity, and high social costs because of the increase of the risk of aspiration pneumonia, other than dehydration, malnutrition [1]. There is no definitive treatment for neurogenic dysphagia. Bath et al. conducted a prospective single-arm, multicenter observational cohort study, treating with PES 245 patients with neurogenic dysphagia [2]. Participants were divided into five groups: 1) stroke not requiring mechanical ventilation; 2) stroke requiring mechanical ventilation and tracheotomy; 3) mechanical ventilation in non-stroke, non-traumatic brain injury (TBI); 4) TBI with or without needing of mechanical ventilation and tracheotomy; and 5) any other neurological cause without the need for mechanical ventilation and tracheotomy. The treatment consisted in stimulating the pharyngeal wall using a catheter consisting in a nasogastric feeding tube with built-in stimulation electrodes with stimulation provided at 5 hz for 10 min for three consecutive days [2]. The primary outcome measure was the validated dysphagia severity rating scale (DSRS) at 3 months. Secondary outcomes comprised other dysphagia severity measures: the penetration-aspiration scale (PAS) and the functional oral intake scale (FOIS). The results showed that, interestingly DSRS improved in patients with both supratentorial and infratentorial stroke without significant differences between the two pathological conditions. In previously ventilated patients, DSRS improvement was more significant in patients who could be decannulated (*n* = 66) as compared to not decannulated patients (*n* = 33). It is of interest that the magnitude of improvement in both primary and secondary outcome measures was less in TBI than other diagnostic groups. Seventy-four serious adverse events (SAE) occurred in 60 participants with pneumonia the most frequent SAE (9.2%) [2].

Although PES has been shown to improve dysphagia after stroke [3], [4], [5] and MS [6], PHADER provides the first evidence that it may work also in other neurological disorders, including TBI and ventilator-related dysphagia such as critical illness polyneuropathy.

No pharmacological treatment has been shown to be effective in improving neurogenic dysphagia. It has been demonstrated that the recovery of dysphagia after a unilateral stroke is associated with an increase of cortico-pharyngeal excitability in the unaffected hemisphere. These cortical reorganization mechanisms are very likely associated with “cerebral plasticity” [8]. Indeed, it was speculated that the effect of PES in swallowing recovery may be due to an increase or triggering of stimulus-mediated cortical rearrangements [8]. The first experiments on PES were carried out by Hamdy's group [7]. This paradigm applied to dysphagic hemiplegic stroke patients resulted in an improvement of swallowing performances and a reduction in the frequency of aspiration [5].

PES has been demonstrated to be a plausible promise in post-stroke and multiple sclerosis (MS)-associated dysphagia [9]. In a study aimed to evaluate the long-term effect of PES in post-stroke dysphagia, PES demonstrated accelerated swallowing recovery over the first two weeks poststimulation [3,9]. A recent meta-analysis of three randomized controlled trial (RCT) on PES treatment for post-stroke dysphagia concluded that PES is associated to less radiological aspiration and clinical dysphagia, leading to possibly reduced length of stay hospital [3]. A recent RCT on 162 dysphagic patients with subacute ischemic or hemorrhagic stroke, showed that PES was safe but did not show significant superiority in aspiration scores as compared to sham stimulation [10]. However, several factors including the enrollment of patients with mild dysphagia may have contributed to these neutral results [10].

Our group evaluated the PES effect on swallowing recovery in 20 MS patients with severe dysphagia who were randomized to receive 5 Hz “real” PES or “sham” pharyngeal stimulation for 10 min. Patients who received “real” PES showed a significant improvement in all the outcome measures as compared with patients receiving “sham” stimulation [6].

In conclusion, the PHADER cohort study demonstrated the effectiveness, tolerance, and safety of PES in a large sample of patients with dysphagia of different etiologies. The results of this study are exciting and encouraging especially because they extend the use of PES to patients with dysphagia associated with neurological diseases different from those evaluated in the previous PES studies and inspire further research to demonstrate the efficacy of this treatment in a broader sample of neurological disorders including neurodegenerative diseases (not evaluated in the present study). A question that should be answered in future studies with PES is whether this tool is equally effective both in dysphagia in which the oral phase is mainly involved and in dysphagia with prevalent pharyngeal phase involvement and/or in dysphagia associated with upper esophageal sphincter hyperactivity. Another outstanding question regards the side of stimulation catheter position and consequently the site of stimulation.

## Declaration of Competing Interest

Nothing to declare.
